# Welcome to *Clinical and Translational Allergy*

**DOI:** 10.1186/2045-7022-1-1

**Published:** 2011-06-10

**Authors:** Jan Lötvall, Victoria Cardona

**Affiliations:** 1 University of Gothenburg, S-405 30 Gothenburg, Sweden; 2 Hospital Universitari Vall d'Hebron, Passeig de la Vall d'Hebron, 119-129, 08035 Barcelona, Spain

## 

It is with the greatest satisfaction and pride that the European Academy of Allergy and Clinical Immunology (EAACI) today can launch its first open access scientific journal, *"Clinical and Translational Allergy" *(*CTA*). Over many years, EAACI have focused on publishing two major journals, *Allergy *as well as *Pediatric Allergy and Immunology *(*PAI*). However, these two excellent journals alone are limited in their ability to publish a considerable number of articles despite their quality, and therefore are insufficient to provide the required platform for communication of many high quality scientific papers for the EAACI community. Consequently, in the last years it has become evident that new journals need to be established by the Academy. It was also felt that Open Access was crucial for any new EAACI journal, as many funding bodies today require the research to be published in this way, and open access makes published research available to the general public, without any need for subscriptions. A key principle for *CTA *will be to provide a very rapid and fair review process for all submission, and we hope the journal becomes a favourite for your, and the allergy, asthma and immunology community as a whole. Further, all manuscripts accepted for publication in *CTA *will be immediately published online, as the publication date is not at all dependent on publication of a printed issue, as is the case for print journals.

The aim of *CTA *is to provide a platform for the dissemination of original allergy-immunology research, exciting reviews as well as EAACI-developed position papers. Today, at the launch of the journal, one position paper and three exciting reviews are published, and several recently submitted original research papers are under consideration. Firstly, a very long-term effort in developing an EAACI position paper on diagnostic tools in rhinology is published in *CTA *[1]. This publication has taken several years to develop, and is the most comprehensive publication on the topic to date. Three review articles are discussing different interesting aspects and theories in allergy. One, by Woo and Bahna [2], discusses the different types of reactions that can be induced by shellfish ingestion, and the clinical misinterpretations that can evolve. Another review article, by Chaudhary and Marr [3], considers the impact that Aspergillus Fumigatus can have on respiratory disease. Lastly, an overview article by Mattila and colleagues [4], examines the role of respiratory and conjunctival epithelium in airway allergic disease. These fundamentally different topics not only contribute to the literature, but also illustrate the width of topics that *CTA *will publish. Beyond clinical experimental research and epidemiology, *CTA *will certainly also accept any studies using animal models of any allergic process, and immunological research related to allergic disease.

The field of allergy concerns the most common group of diseases globally. Allergic diseases have grown rapidly in prevalence in the industrialized world, and are now rapidly growing in developing countries. It is considered that diseases of the immune system, such as allergic diseases, are best explained through a clear grasp of normal immune mechanisms and the ways by which these processes become dysfunctional. This is one of the explanations of selecting the name of the journal, including the key word "translational", since it is likely that the most interesting future results will evolve from truly translational research. Therefore, manuscripts describing research that utilises several techniques to prove a key biological process, or explaining effect of a treatment, will receive extra attention by the journal.

Each manuscript submitted to *CTA *will be externally peer reviewed, to achieve independent input on the quality of the research, the appropriateness of any review, and the quality of the research presented. The written English will also need to be of very high quality for any manuscript to be considered by the journal. Importantly, the Editor's decisions remain independent, and are not only based on reviewer's comments and suggestions, but also on priorities made by the Editors. The current editorial board of *CTA *has primarily been recruited among leaders within the EAACI community (http://www.ctajournal.com/about/edboard). However, applications for membership in the Editorial Board are welcome by e-mail, including an attached Curriculum Vitae and specified field of interest.

One advantage with the open access system is the availability of the published research online. However, any publication process costs money, and since there is no subscription income to open access journals, the cost will have to be covered by the publishing authors. Therefore, *CTA *will charge a nominal fee for any article published, which will have to be covered by the authors of the article. This is becoming a common approach for many scientists preferably publishing in Open Access journals, and the total cost for publishing in *CTA *is lower than for subscriptions to other journals. The nominal fee for publishing in *CTA *is currently £1095 (€1250). As for all BioMed Central journals, authors from countries listed in the World Bank low or lower-middle income bracket are automatically granted a waiver of the publication charges, to ensure that inability to pay is not a barrier to publication [5].

Last, but not least, *CTA *is established not only for the benefit of EAACI, but for the good of the global allergy community. Again this is well illustrated by the first published reviews [1-4], including authors from many parts of the world. We look forward to receiving your submission in the not too distant future, regardless of which continent you live in.

Jan Lötvall, Editor-in-Chief - *Clinical and Translational Allergy*, Current EAACI President

Victoria Cardona, Managing Editor - *Clinical and Translational Allergy*, Current EAACI Vice President Communication and Membership
